# Assessing the cardiovascular and potassium lowering effects of levalbuterol compared to albuterol: a randomized control trial

**DOI:** 10.3389/fcvm.2025.1463999

**Published:** 2025-12-08

**Authors:** Baraa Shebli, Mike Ghabally, Muhammad Besher Shabouk, Ahmad Yamen Arnaout, Mohamed Besher Zeina, Mahmoud Malhis

**Affiliations:** 1 Faculty of Medicine, Department of Internal Medicine, Division of Cardiology, University of Aleppo, Aleppo University Hospital, Aleppo, Syria; 2 Faculty of Medicine, University of Aleppo, Aleppo University Hospital, Aleppo, Syria; 3 Faculty of Medicine, Department of Internal Medicine, University of Aleppo, Aleppo University Hospital, Aleppo, Syria; 4 Department of Mathematical Statistics, Faculty of Science, University of Aleppo, Aleppo, Syria

**Keywords:** levalbuterol, albuterol, hyperkalemia, cardiovascular side effects, beta2-agonists

## Abstract

**Background:**

Beta-agonists like Levalbuterol and Albuterol are used in the treatment of Hyperkalemia. However, few studies in the medical literature have directly compared the cardiac effects and potassium-lowering efficacy of levalbuterol and albuterol, yielding inconclusive results. Understanding the cardiovascular effects and potassium-lowering abilities of these medications in hyperkalemic patients is crucial for optimizing treatment strategies and minimizing adverse events.

**Methods:**

This randomized controlled trial conducted at Aleppo University Hospital in Syria aimed to compare the cardiovascular and potassium-lowering effects of Levalbuterol vs. Albuterol in hyperkalemic patients. The study, carried out between October 2021 and February 2022, utilized a single-center single-blinded two-armed design with patients randomized using a computer-generated sequence and block randomization method.

**Results:**

The results showed no significant difference in mean HR change between Levalbuterol and Albuterol groups at 30 min, nor in the change in potassium serum levels at 90 min. Both drugs exhibited similar patterns in HR changes over time and blood pressure variations at different points. Despite limitations in data collection for side effects, the reported symptoms in both groups were consistent with known side effects of beta-agonists, with tremors and nervousness being the most commonly reported.

**Conclusion:**

Our study provides the first direct insights into the effects of Levalbuterol and Albuterol on heart rate, potassium levels, and blood pressure which would be very important in specific cases such as in patients with heart failure. Both medications showed similar patterns in heart rate changes, potassium levels, and blood pressure variations at different time points.

**Clinical Trial Registration:**

Clinicaltrials.gov, identifier NCT05173584.

## 1 Introduction

Hyperkalemia, a common and life-threatening electrolyte disturbance, poses significant risks by impairing cardiac and systemic organ functions. Despite the severity of this condition, a definitive treatment guideline for hyperkalemia in emergency settings remains elusive. The recent “kidney disease: Improving Global Outcomes (KDIGO)” conference provided a treatment protocol for hyperkalemia, shedding light on the existing controversies in this area. Among the recommended approaches is the use of beta2-agonists, a key component in managing hyperkalemia (1, 2). However, the evidence supporting this treatment strategy is limited to racemic albuterol.

Salbutamol, also known as albuterol, is a medication commonly used to treat asthma and chronic obstructive pulmonary disease (COPD). It is comprised of two enantiomers, R-albuterol (levosalbutamol or levalbuterol) and S-albuterol. Levalbuterol is the isolated R-enantiomer of racemic albuterol. Enantioselective pharmacology suggests that the (R)-enantiomer is responsible for the desired bronchodilatory and potassium-lowering effects via β2-adrenergic receptor agonism, while the (S)-enantiomer may be pharmacologically active, potentially promoting pro-inflammatory pathways and contributing to adverse effects. This has led to the hypothesis that levalbuterol may have an improved therapeutic profile compared to the racemic form. Studies have postulated that the cardiac side effects associated with albuterol could be attributed to the S-enantiomer, while the R-enantiomer may have a more favorable safety profile. However, evidence directly supporting this theory is limited, as few studies have compared the cardiac effects and potassium-lowering efficacy of levalbuterol and albuterol, yielding inconclusive results (3–6).

Given this research gap, our study aims to assess the cardiovascular and potassium-lowering effects of levalbuterol compared to albuterol in hyperkalemic patients using appropriately adjusted dosing regimens. To the best of our knowledge, no prior study has comprehensively evaluated the efficacy and safety of these two bronchodilators in hyperkalemic patients. Our single-centered controlled clinical trial, conducted at Aleppo University Hospital, seeks to provide valuable insights into the potential of levalbuterol as a substitute for albuterol in lowering potassium levels while minimizing cardiac side effects in patients with hyperkalemia.

## 2 Methods

### 2.1 Participants and allocation

Our study was a single-center single-blinded two-armed randomized controlled trial conducted at Aleppo University Hospital in Aleppo, Syria. The main goal of the trial was to assess the cardiac side effects of Albuterol vs. Levalbuterol in hyperkalemic patients. The study was conducted between October 2021 and February 2022.

We included all patients older than 16 years presenting to the emergency department of Aleppo University Hospital or inpatients in the hospital wards with hyperkalemia >5.9 mEq/L, either symptomatically or incidentally. We excluded patients under the age of 16, pregnant patients, those with hypersensitivity to the medications, diabetic patients presenting with diabetic ketoacidosis or hyperosmolar hyperglycemic state, hyperthyroid patients, patients with hemodynamic instability, those expected to require emergency intubation and ventilation, and those expected to require hemodialysis within the next 60 min. We also excluded patients on pacemakers, beta-blockers, beta-agonists, or any anti-arrhythmic drug, and those with pathologic arrhythmias, baseline tachycardia >120 bpm, acute exacerbation of heart failure, or acute coronary syndrome.

We included patients currently taking furosemide since furosemide's effect on potassium in the acute setting is minor and no study to date has examined its efficacy (7). We also included patients who had been administered sodium bicarbonate -when indicated- as it does not have a significant acute effect on serum potassium (5). As insulin has a lowering effect on serum potassium levels, we intended in our protocol to exclude all type 1 diabetes patients on insulin. However, 90% of insulin would have been eliminated after 3 and 1/3 of its half-life (8) and its effect on potassium levels would be negligible. Therefore, patients whose last dose passed 3 and 1/3 of its half-life were included in the study.

Each lab value that showed hyperkalemia was repeated for confirmation. Lab samples that were suspected to have pseudohyperkalemia were excluded. We considered the following cases of pseudohyperkalemia: hemolysis of blood sample, thrombocytosis > 10^6^/mm^3^, leukocytosis > 10^5^/mm^3^, mechanical trauma during venipuncture, fist clenching during blood drawing, or tourniquet time > 1 min.

Patients were randomized using a computer-generated randomization sequence. To avoid possible imbalances, we used block randomization method with a block of four. We stratified patients according to the presence of end-stage renal disease. Patients were blinded to the treatment they received. The healthcare providers administering the interventions were not blinded due to logistical constraints; however, all outcome assessors (laboratory personnel and electronic vital sign monitors) were blinded to the treatment allocation, minimizing assessment bias for the primary outcomes.

### 2.2 Intervention details

Included participants received either nebulized Albuterol 10 mg or nebulized Levalbuterol 5 mg in distilled water along with other standard hyperkalemia medications if needed. All patients received an equivalent of 10 IU Insulin renal-adjusted with 25 g Glucose in a concentration convenient to the volume status of the patient. Insulin dosage was adjusted in case of decreased eGFR. We administered only 75% of the dose if eGFR is <50% and 50% of the dose if eGFR is <10 (9, 10). Patients with potassium higher than 6.5 mEq or with ECG changes associated with hyperkalemia were given calcium gluconate 30 mg to stabilize cardiac cell membranes (11).

We used a paper form to document the baseline characteristics of each patient along with their chief complaint -if any-, vital signs, current medications, current symptoms, lab tests, drugs given in the acute setting, and adverse reactions. Lab tests included serum levels of all of the following: potassium (baseline, confirmation, and after 90 min), and baseline sodium, glucose, creatinine, urea, white cell counts, hemoglobin, and platelets. Heart rate was documented at baseline, 15 min, 30 min, 45 min, 60 min, 75 min, 90 min, and 120 min. Blood pressure was documented at baseline, 30 min, 60 min, and 90 min.

The paper forms were filled out by resident physicians trained in a workshop before the trial began. Data from the paper forms were uploaded into Google Forms and exported to an Excel file. Senior resident physicians reviewed collected forms daily to check progress and data integrity.

### 2.3 Ethical approval and protocol registration

The protocol of this trial was reviewed and approved by the institutional review board at the University of Aleppo and was formed according to the code of ethics announced in the Declaration of Helsinki and its later amendments. Our protocol was registered in Clinicaltrials.gov with an identifier of NCT05173584 and in the central registry of the University of Aleppo with a serial number of 15,993 class 1/14.

### 2.4 Study outcomes

Our primary outcomes were the HR change between baseline and 30 min after drug administration and the change in potassium serum levels between baseline and 90 min after drug administration. This time point of 30 min was selected based on multiple studies that consistently reported it as the time of greater difference in HR between levalbuterol and racemic albuterol (12, 13). Secondary outcomes included HR changes at 15, 30, 45, 60, 75, 90, and 120 min after nebulization, and change in systolic and diastolic blood pressure at 30, 60, and 90 min after nebulization. Baseline characteristics of included patients were documented and compared between the two treatment groups.

### 2.5 Sample size calculation

The sample size was calculated to detect a 5.0% increase in the mean of the continuous endpoint between Group 1 (albuterol, mean assumed = 4.4) and Group 2 (levalbuterol, mean assumed = 4.625), based on a previous study (13). Using a significance level (alpha) of 0.05 and a power (1-β) of 0.8, a sample size of 13 per group (total *n* = 26) was required. This calculation was performed using power analysis. We decided to enroll a larger sample than required to account for potential dropouts; however, the final sample size remained limited, which is acknowledged as a study limitation.

### 2.6 Statistical analysis

We used as-treated analysis instead of intention-to-treat as we are interested in studying the adverse effects of the drugs. We performed descriptive statistics to describe patients' characteristics classified by treatment group. We reported categorical data as frequencies; then, we compared the groups using the Chi-square test. Continuous outcomes were compared between the two distinct treatment groups using the independent t-test. All data analyses were done using IBM SPSS (Version 29).

## 3 Results

**Figure 1:**
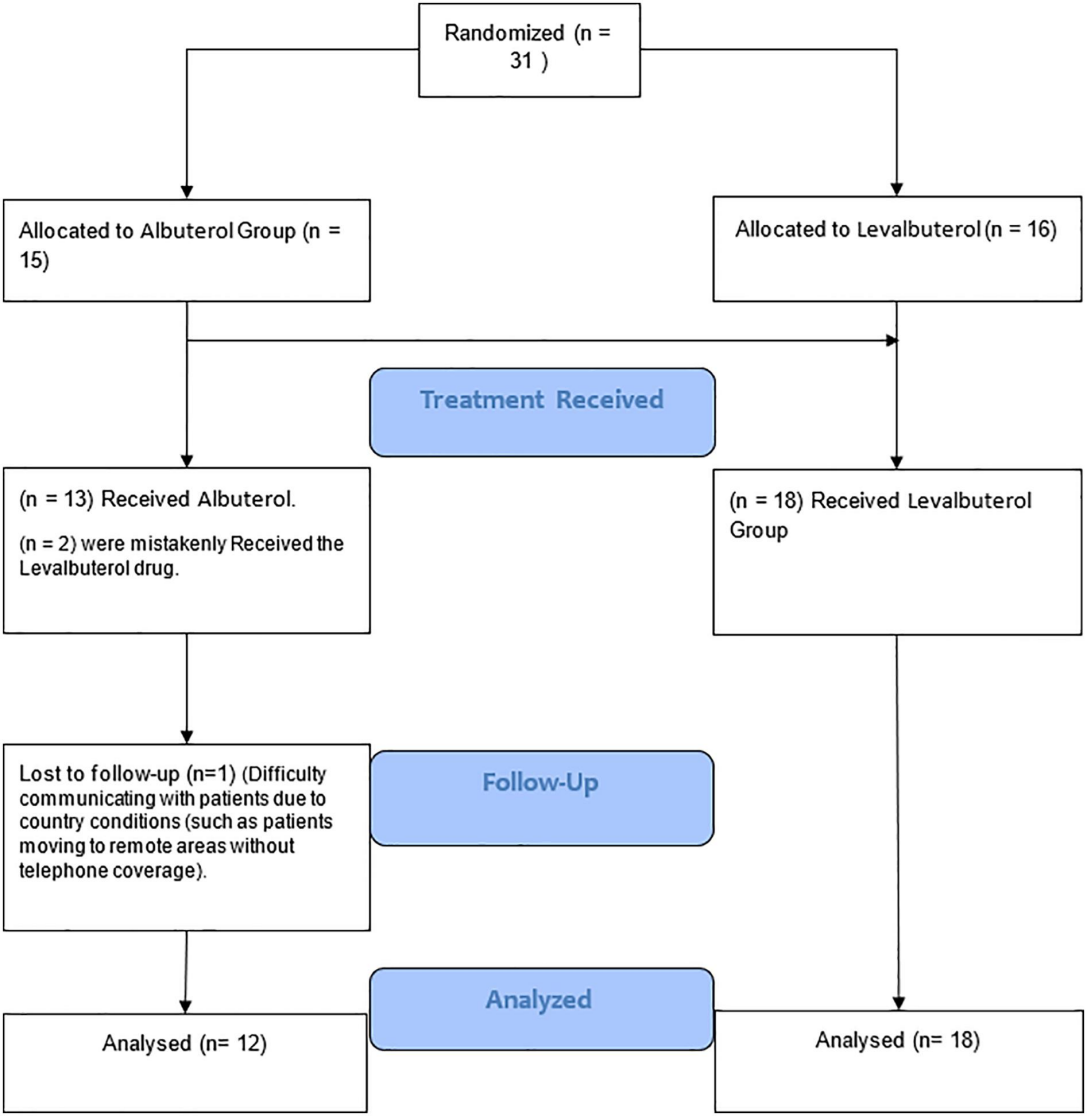
Flow chart of the study.

The trial initially included 31 patients (15 albuterol, 16 levalbuterol). We lost data for one patient in the albuterol group, and two patients were mistakenly given levalbuterol. Consequently, 30 patients remained for analysis: 18 (60%) in the levalbuterol group and 12 (40%) in the albuterol group (Figure 1). Baseline characteristics of the population are shown in Table 1. Sixteen (53.3%) patients were men and 14 (46.7%) were women. The groups were homogeneous in terms of sex and age. Mean eGFR, HR, and serum potassium levels at baseline showed no significant differences. Mean baseline potassium levels were 6.6 mEq/L (SD = 0.46) in the levalbuterol group and 6.7 mEq/L (SD = 0.43) in the albuterol group (*p* > 0.05). The most common comorbidities were CKD (50%) and hypertension (50%). There were differences in baseline medications: ACEi/ARBs/ARNI (levalbuterol 62.5% vs. albuterol 25%), potassium-sparing diuretics (levalbuterol 31.25% vs. albuterol 8.33%), and thiazide diuretics (levalbuterol 31.25% vs. albuterol 8.33%). Hypertension was more prevalent in the levalbuterol group (64.71% vs. 33.3%). The distribution of end-stage renal disease patients was equal between groups.

**Table 1:** Baseline characteristics of the patients.

Characteristic	Levalbuterol (*n* = 18)	Albuterol (*n* = 12)	*P* -value
Sex - Male (*n*, %)	(9, 50%)	(7, 58.33%)	0.654
Age (years) (mean, SD)	(53.89, 16.47)	(58.17, 19.51)	0.538
Comorbidities			
CKD (*n*, %)	(8, 47.06%)	(7, 58.33%)	0.550
Hypertension (*n*, %)	(11, 64.71%)	(4, 33.33%)	0.096
T2DM (*n*, %)	(7, 41.18%)	(4, 33.33%)	0.668
ESRD (*n*, %)	(9, 50%)	(8, 66.67%)	0.367
Confirmation potassium level (mEq) *n* (mean, SD)	(6.59, 0.46)	(6.68, 0.43)	0.591
Heart rate (bpm) *n* (mean, SD)	(91.06, 18.71)	(93.23, 13.97)	0.719
eGFR *n* (mean, SD)	(19.67, 19.76)	(16.67, 18.09)	0.688
Systolic blood pressure mmHg *n* (mean, SD)	(136, 26.86)	(134.17, 26.01)	0.853
Diastolic blood pressure mmHg *n* (mean, SD)	(72.78, 16.74)	(75.83, 14.28)	0.597
Hemoglobin (g\dl) *n* (mean, SD)	(8.89, 2.50)	(9.73, 2.14)	0.396
Medications			
Beta-blockers (*n*, %)	(2, 12.50%)	(2, 16.67%)	0.755
ACEi (*n*, %)	(2, 12.50%)	(0, 0%)	0.223
ARBs (*n*, %)	(7, 43.75%)	(3, 25%)	0.306
ARNI (*n*, %)	(1, 6.25%)	(0, 0%)	0.378
NSAIDs (*n*, %)	(2, 12.50%)	(1, 8.33%)	0.724
Potassium-sparing diuretics (*n*, %)	(5, 31.25%)	(1, 8.33%)	0.144
Loop diuretics (*n*, %)	(5, 31.25%)	(4, 33.33%)	0.907
Thiazide diuretics (*n*, %)	(5, 31.25%)	(1, 8.33%)	0.144

CKD, chronic kidney disease; T2DM, type 2 diabetes mellitus; ESRD, end-stage renal disease; eGFR, estimated glomerular filtration rate; bpm, beats per minute.

The change in heart rate at 30 min and the change in potassium serum levels at 90 min can be seen in Table 2. Mean potassium change was lower in the levalbuterol group −0.74 (SD = 0.45) than in the albuterol group −1.31 (SD = 1.19) [*p*-value = 0.176]. On the contrary, there was no significant difference in mean heart rate change between the two groups; levalbuterol was 12.0 (SD = 11.42) and albuterol was 17.27 (SD = 10.66) [*p*-value = 0.214].

**Table 2:** Primary outcomes by treatment group.

Outcome	Drug name	*N*	Mean	SD	*P*-value
HR change at 30 min^a^	Levalbuterol	18	12.00	11.42	0.214
	Albuterol	12	17.27	10.66	
*K* + level change at 90 min^b^	Levalbuterol	15	−0.74	0.45	0.176
	Albuterol	10	−1.31	1.19	

a HR change from baseline.

b Potassium change from confirmation lab test.

**Figure 2:**
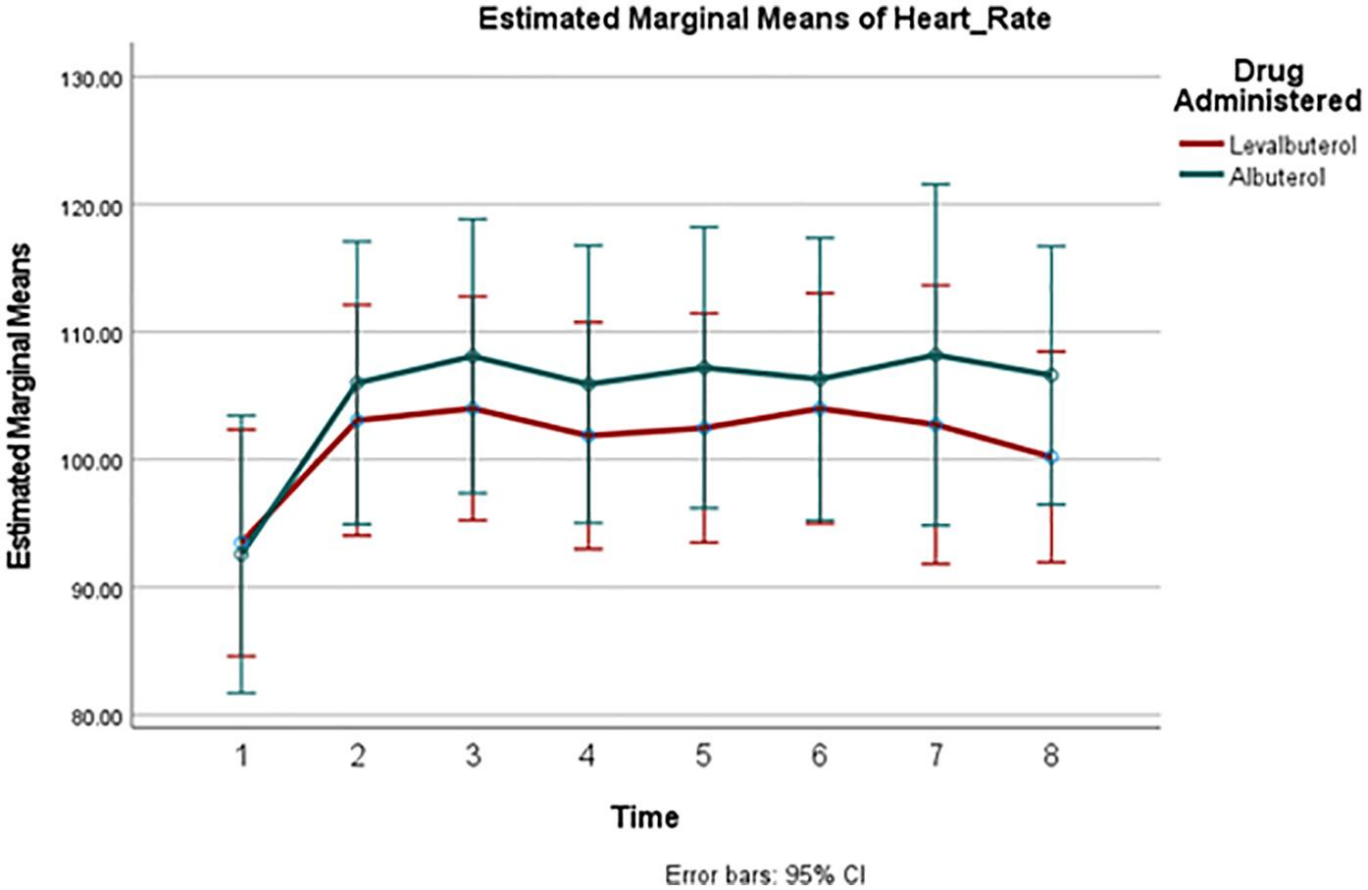
Mean HR change over time. *Y*-axis (Vertical): “Heart Rate (beats per minute, bpm)”. *X*-axis (Horizontal): “Time After Drug Administration (minutes)” Point 1: Baseline, Point 2: 15 min, Point 3: 30 min, Point 4: 45 min, Point 5: 60 min, Point 6: 75 min, Point 7: 90 min, Point 8: 120 min.

**Figure 3:**
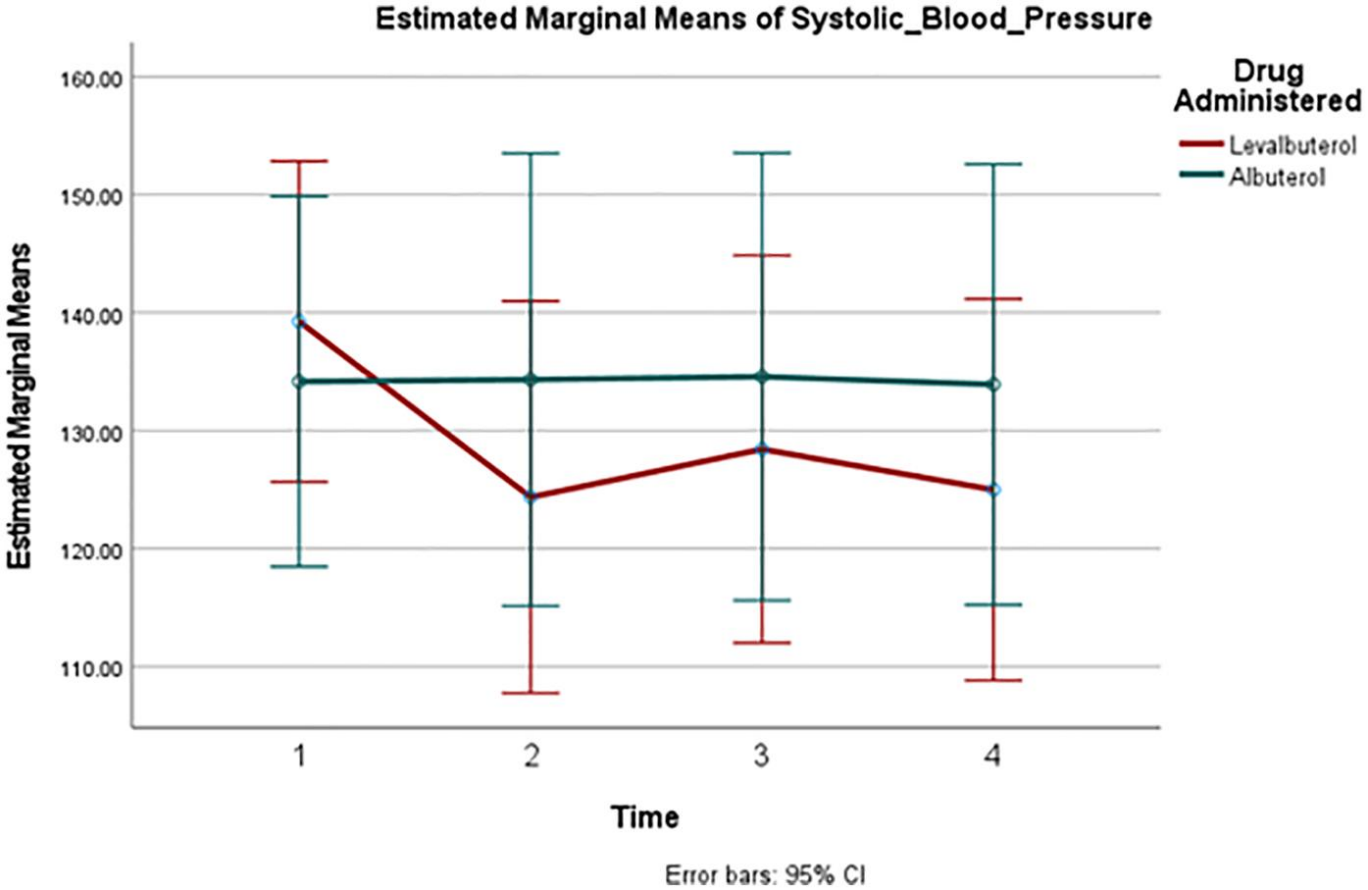
Mean systolic blood pressure change over time. Point 1: Baseline, Point 2: 30 min, Point 3: 60 min, Point 4: 90 min.

**Figure 4:**
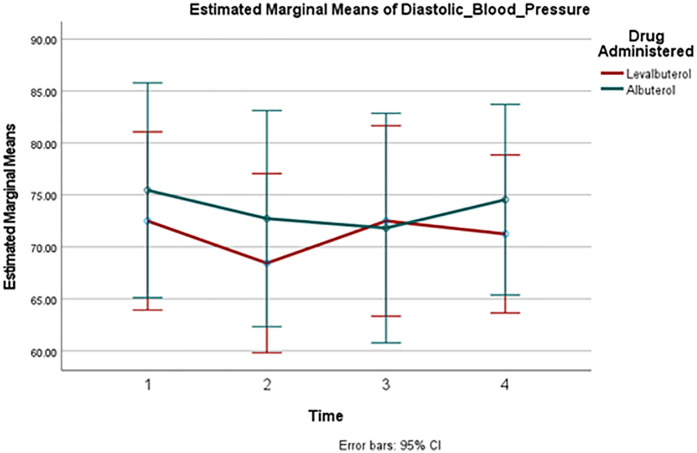
Mean diastolic blood pressure change over time. Point 1: Baseline, Point 2: 30 min, Point 3: 60 min, Point 4: 90 min.

A comparison of HR changes over time between the two groups can be seen in Figure 2. Despite the well-observed rise in heart rate in both drugs, there was no significant difference in these changes between the two groups at any measured point. Figure 3 and Figure 4 track the changes in systolic and diastolic blood pressure respectively over time. Neither pressure differed significantly between the two groups at any given point.

Regarding side effects, a reasonable amount of data was lost in the levalbuterol group compared to the albuterol group. Therefore, the remaining data were not sufficient to do analysis. Among the twelve patients in the albuterol group, there were: five incidents of tremor, five nervousness, three palpitations, two nausea or vomiting, one hypoglycemia, and one headache. None of them had hypokalemia, lightheadedness, or fever. Eleven out of the 18 patients in the levalbuterol group had adverse effects data. Among those 11 patients, three patients reported tremors, four reported nervousness, two reported palpitations, three reported nausea or vomiting, one had a headache, one had hypokalemia, and one had a fever. None of them complained of lightheadedness or hypoglycemia.

## 4 Discussion

The results of our study comparing the effects of Levalbuterol and Albuterol on heart rate and potassium levels in patients with heart failure and comorbidities reflect some interesting findings. The baseline characteristics of the patients were similar in terms of age, sex, and key clinical parameters, ensuring a fair comparison between the groups.

The enantioselective pharmacology of β2-agonists suggests a potential for improved safety with levalbuterol (6, 14). However, there is limited evidence supporting this concept, given the scarcity of studies that directly compared the cardiac effects of levalbuterol vs. albuterol, resulting in inconclusive findings (12, 13, 15). In our study, the changes in heart rate did not show a significant discrepancy between the two groups. While heart rate increased in both groups, the magnitude of change was similar. In another study comparing heart rate changes associated with levalbuterol and racemic albuterol in pediatric cardiology patients, it was found that racemic albuterol and levalbuterol were associated with increased heart rate. This increase was found to be equivalent, with both medications causing a mean increase in heart rate of 6.8 beats/min and 6.2 beats/min, respectively (12).

After evaluating the change in potassium levels between Levalbuterol and Albuterol, we observed a noteworthy difference in the mean potassium change, suggesting that Levalbuterol might have a lesser impact on potassium levels despite the lack of statistical significance. This finding is consistent with the conclusion drawn from the review of seven studies indicates that nebulized levalbuterol appears to be as effective as albuterol in lowering serum potassium in adults (16). Moreover, Diana Pancu et al. conducted a randomized, double-blind, placebo-controlled prospective study comparing the K + -lowering effects of nebulized saline, albuterol, and levalbuterol in healthy adult volunteers. The results of their study show that levalbuterol is as effective as racemic albuterol in lowering serum potassium, with levalbuterol causing fewer adverse effects as reported (17). Supporting the dose-dependent potassium-lowering effect of beta2-agonists, a recent crossover study in healthy dogs demonstrated that inhaled albuterol significantly decreased blood potassium concentrations in a dose-dependent manner without causing clinically meaningful tachycardia or hyperglycemia (18).

The incidence of side effects was notable in both groups, with a higher proportion of patients in the Levalbuterol group experiencing adverse effects. Despite limitations in data collection for side effects, the reported symptoms in both groups were consistent with known side effects of beta-agonists, with tremors and nervousness being the most commonly reported. In a systematic review and meta-analysis comparing Levalbuterol vs. albuterol for acute asthma, it was found that there were no significant differences in side effects between the two groups (19).

The lack of significant differences in blood pressure changes between the groups at various time points suggests that both drugs have a similar impact on blood pressure regulation in these patients. This finding is important in guiding clinical decision-making regarding the choice of bronchodilators in heart failure patients with comorbidities.

The limitations of our study include the small sample size and the heterogeneous nature of the patient population. Additionally, the limited generalizability of the results due to the study being conducted at a single center in Aleppo, Syria, may restrict the applicability of the findings to a broader population. While patients were blinded to their treatment, the healthcare providers administering the interventions were not blinded. However, this lack of blinding did not affect the main outcome results because they were recorded using laboratory tests or electronic devices. We deliberately excluded certain subgroups of patients, including individuals with baseline tachycardia or acute coronary syndrome, to ensure that the study results would provide a precise and accurate response to the research question regarding cardiac effects.

## 5 Conclusion

Our study provides the first direct insights into the effects of Levalbuterol and Albuterol on heart rate, potassium levels, and blood pressure in patients with heart failure. Both medications showed similar patterns in heart rate changes, potassium levels, and blood pressure variations at different time points. Further research may be warranted to explore differences in clinical outcomes or side effect profiles between Levalbuterol and Albuterol in this patient population.

## Data Availability

The raw data supporting the conclusions of this article will be made available by the authors, without undue reservation.

## Aleppo University Hospital Team

Ayman Abbas; Ahmed Mustafa; Ayham Zahran; Amer Obeid; Bashar Raji; Abdulmonem Nahar; Mohammad Tantoura
